# Impact of intracellular innate immune receptors on immunometabolism

**DOI:** 10.1038/s41423-021-00780-y

**Published:** 2021-10-25

**Authors:** Wei-Chun Chou, Elena Rampanelli, Xin Li, Jenny P.-Y. Ting

**Affiliations:** 1 Lineberger Comprehensive Cancer Center, University of North Carolina at Chapel Hill, Chapel Hill, NC 27599 USA; 2 Department of Genetics, University of North Carolina at Chapel Hill, Chapel Hill, NC 27599 USA; 3 Amsterdam UMC (University Medical Center, location AMC), Department of Experimental Vascular Medicine, AGEM (Amsterdam Gastroenterology Endocrinology Metabolism) Institute, Meibergdreef 9, 1105 AZ Amsterdam, The Netherlands; 4 Comparative Immunology Research Center, College of Veterinary Medicine, China Agricultural University, Beijing, 100193 China

**Keywords:** Immunometabolism, NLRs, NLRP3/AIM2 inflammasomes, innate sensors/receptors, STING, AKT-mTOR, Immunology, NOD-like receptors

## Abstract

Immunometabolism, which is the metabolic reprogramming of anaerobic glycolysis, oxidative phosphorylation, and metabolite synthesis upon immune cell activation, has gained importance as a regulator of the homeostasis, activation, proliferation, and differentiation of innate and adaptive immune cell subsets that function as key factors in immunity. Metabolic changes in epithelial and other stromal cells in response to different stimulatory signals are also crucial in infection, inflammation, cancer, autoimmune diseases, and metabolic disorders. The crosstalk between the PI3K–AKT–mTOR and LKB1–AMPK signaling pathways is critical for modulating both immune and nonimmune cell metabolism. The bidirectional interaction between immune cells and metabolism is a topic of intense study. Toll-like receptors (TLRs), cytokine receptors, and T and B cell receptors have been shown to activate multiple downstream metabolic pathways. However, how intracellular innate immune sensors/receptors intersect with metabolic pathways is less well understood. The goal of this review is to examine the link between immunometabolism and the functions of several intracellular innate immune sensors or receptors, such as nucleotide-binding and leucine-rich repeat-containing receptors (NLRs, or NOD-like receptors), absent in melanoma 2 (AIM2)-like receptors (ALRs), and the cyclic dinucleotide receptor stimulator of interferon genes (STING). We will focus on recent advances and describe the impact of these intracellular innate immune receptors on multiple metabolic pathways. Whenever appropriate, this review will provide a brief contextual connection to pathogenic infections, autoimmune diseases, cancers, metabolic disorders, and/or inflammatory bowel diseases.

## Introduction

**Fig. 1:**
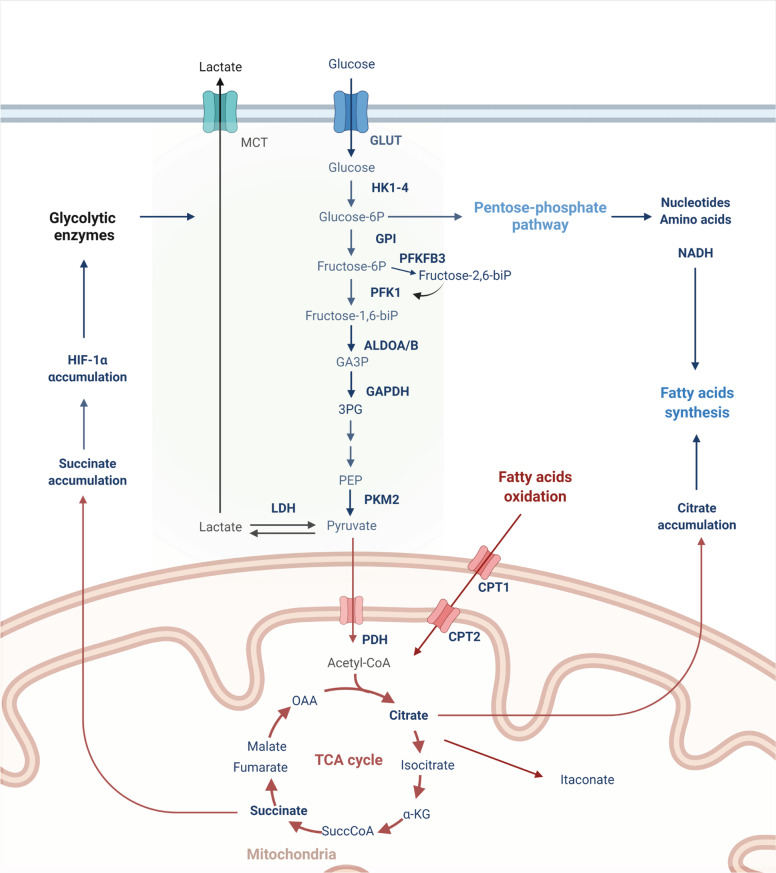
Glycolysis (light green box) supports the anabolic demands of activated immune cells. Upon immune activation, glycolysis shifts pyruvate toward lactate production instead of feeding the TCA cycle. Accumulation of the TCA intermediate succinate promotes glycolysis via HIF-1α stabilization. In addition, the accumulated citrate serves as a precursor for the anti-inflammatory metabolite itaconate. Glycolysis also fuels the pentose phosphate pathway (PPP) to generate nucleotides, amino acids, and NADPH, which is used with citrate for fatty acid synthesis. Oxidized fatty acids drive mitochondrial oxidative metabolism, which is more prominent in tolerogenic immune cells than in other cells

A growing number of studies have highlighted the intricate relationship between intracellular metabolism and inflammation. The immunometabolism field focuses on the alterations in intracellular metabolism that accompany immune cell activation and control immune cell functions. Upon activation, immune cells undergo extensive metabolic reprogramming to fulfill the drastic increase in energy demand and support immune cell functions, such as robust cytokine production, rapid proliferation, and migratory activities [1]. In particular, the increased requirement for glucose and the upregulation of the rate of aerobic glycolysis are key components of the intracellular metabolic switch induced by proinflammatory signals, which lead to a shift in glucose metabolism toward the production of lactate rather than the mitochondrial tricarboxylic acid (TCA) cycle (Fig. 1). Although extensive studies have shown the importance of metabolic reprogramming in both T and B cell receptor (TCR/BCR) signaling, as well as membrane-associated Toll-like receptors (TLRs), less is known about the role of intracellular innate immune receptors in immunometabolism. The purpose of this review is to highlight some key findings that reveal how innate immune receptor signaling intersects with immunometabolism.

In immunometabolism, the mitochondrial TCA cycle is coupled to oxidative phosphorylation (OXPHOS) and is a highly effective energy generation process; however, glycolysis can be more rapidly enhanced to meet the energetic demands of activated proinflammatory immune cells [2] (Fig. 1). This enhancement occurs via the upregulation of glycolytic enzymes, such as hexokinase 1 and 2 (HK1, HK2), glyceraldehyde-3-phosphate dehydrogenase (GAPDH), and pyruvate kinase isoenzyme M2 (PKM2), and via an increase in the surface expression of the glucose transporter GLUT1 [3–6]. Moreover, glycolysis fuels the pentose phosphate pathway (PPP), which amplifies the production of glycolysis-derived intermediates and is crucial in supporting proliferation and immune effector functions. In fact, the PPP generates biosynthetic precursors for nucleotides, amino acids, and fatty acid synthesis (FAS), thereby supporting anabolic growth and cytokine secretion [1, 7]. Furthermore, the NADPH produced in the PPP is used for the rapid production of microbicidal reactive oxygen species (ROS) by NADPH oxidase, as well as for glutathione biosynthesis, which counteracts oxidative stress [8].

Engagement of immune receptors such as TLRs, TCR/BCR, or interleukin-2 receptor (IL-2R) dictates the metabolic switch in immune cells. Notably, these immune receptors activate the NF-κB (nuclear factor kappa-light-chain-enhancer of activated B cells) pathway and the transcription factors HIF-1α (hypoxia-inducible factor-1α) and c-Myc, thereby inducing transcriptional reprogramming toward glycolytic gene expression [3, 9–11]. Additionally, receptor-mediated activation of kinase signalings, such as the Akt (also known as protein kinase B) and mTOR (mechanistic target of rapamycin) pathways, largely mediates the high demand for cellular nutrients by sustaining glucose uptake and glycolysis, inducing de novo synthesis of fatty acids, lipids, and cholesterol, and promoting glutamine anaplerosis and protein translation [12]. mTOR is a master regulator of intracellular metabolism and immune cell activation and constitutes the catalytic subunit of the distinct multiprotein complexes mTORC1 and mTORC2 (mTOR complexes 1 and 2, respectively). mTORC1 is activated by the phosphatidylinositol 3-kinase (PI3K)/Akt pathway, whereas mTORC2 controls Akt activation by inducing Akt phosphorylation. Both complexes contribute to metabolic reprogramming: mTORC1 facilitates glycolysis, glutaminolysis, and protein and lipid synthesis and inhibits autophagy, while mTORC2 activates Akt, which promotes GLUT1 surface expression and aerobic glycolysis. By modulating downstream effectors, such as S6K1, 4EBP1, and Akt, these complexes regulate cell growth, proliferation, and aerobic glycolysis in response to growth factors, nutrients, and receptor signaling [12–14].

The marked dependence on glycolysis has been largely studied in classically activated M1 macrophages, monocytes, and effector T-helper 1 and 17 (Th1/17) cells [3, 6, 9, 11, 15], but glycolysis is a hallmark of all activated immune cells, including activated dendritic cells (DCs), neutrophils, B cells, and natural killer cells [9, 16–19]. Conversely, energy production via OXPHOS is more prominently associated with the longevity of quiescent homeostatic immune cells, such as naïve and memory B/T cells [20–23], and cells with immunotolerogenic properties, such as alternatively activated M2 macrophages and regulatory (Treg) or exhausted T cells [24–27]. Mitochondrial oxidative metabolism is fueled by aerobic glycolysis through the conversion of the glycolytic end-product pyruvate into acetyl-CoA and by mitochondrial fatty acid oxidation (FAO), which generates large amounts of acetyl-CoA, NADH, and FADH_2_ [1]. Acetyl-CoA is used in the TCA cycle; NADH and FADH_2_ are used in the electron transport chain (ETC) to produce ATP [1]. Mitochondria-dependent catabolic metabolism is largely regulated by AMPK (AMP-activated protein kinase), which is a central regulator of energy balance that is activated by energy stress (such as low AMP/ADP-to-ATP ratio, glucose/glutamine deprivation) and by the kinases LKB1 (liver kinase B1) and CaMKK2 (calcium calmodulin kinase 2). AMPK favors cellular catabolism by promoting mitochondrial fitness and suppressing anabolic processes [28]. Moreover, AMPK drives mitochondrial biogenesis and fission, as well as the clearance of damaged mitochondria via mitophagy/autophagy. Conversely, AMPK inhibits the enzymes acetyl-CoA carboxylase and HMG-CoA reductase, which are crucial in lipid and cholesterol synthesis, respectively, and inhibits mTORC1 activity [28, 29]. Hence, by suppressing anabolism and mTOR, AMPK inhibits metabolic rewiring and limits immune cell activation.

Adding to the complexity of this intricate immunometabolic balance, the choice of energy source serves specialized immune phenotypes and specific immune functions. For example, Tregs are less reliant on glycolysis than Th1/2/17 cells, although they use glycolysis to meet the energetic demands required for migration, and glycolysis complements oxidative metabolism during expansion [30, 31]. In addition, glycolysis was shown to enhance Foxp3 (a master regulator of Treg differentiation and immunotolerogenic functions) splicing variants containing exon 2 through the glycolytic enzyme enolase-1 [32]. Similarly, while tolerogenic M2 macrophages and Tregs are associated with increased oxidative metabolism and FAO rates, recent studies using cell-specific genetic depletion of the *Cpt1a* gene, which encodes the mitochondrial long-chain FA transporter carnitine palmitoyl transferase-1, have revealed that FAO is dispensable for M2 macrophages or Treg differentiation, disputing previous results obtained with the use of the CPT1 inhibitor etomoxir [6, 33–36].

During proinflammatory immune activation, the rapid increase in glycolytic flux at the expense of OXPHOS not only supports the generation of energy and biomass but also results in the accumulation of metabolic intermediates, such as succinate, itaconate, and fumarate, which may function as immunoregulatory signaling molecules (immunometabolites) [37–40]. Moreover, this metabolic repurposing has been shown to notably reshape the functional status of immune cells, particularly innate cells, through epigenetic reprogramming. The latter is tightly influenced by changes in the network of intracellular metabolic pathways and, thus, the bioavailability of metabolic intermediates, such as acetyl-coenzyme A, NAD^+^, and α-ketoglutarate, which act as enzymatic cofactors of histone acetyltransferases, deacetylases, and histone-/DNA-demethylases, respectively [40].

Overall, a better understanding of the kinetics of immune-metabolic circuits mediated by innate immune receptor engagement can provide novel therapeutic opportunities to either boost immune defenses against pathogen infections or harness excessive pathologic inflammation in chronic inflammatory metabolic diseases. Next, we will examine the impact of several intracellular innate immune receptors/sensors (NLRs, AIM2, and STING) and immunometabolism.

## NLRs and immunometabolism

The NLR (nucleotide binding domain leucine-rich repeat or nucleotide-oligomerization domain (NOD)-like receptors) family is a large group of evolutionarily conserved sensors or receptors that recognize pathogens and homeostatic alterations [41]. All members have a nucleotide-binding domain and a leucine-rich repeat (LRR) domain. Members have divergent functions including the transcriptional activation of major histocompatibility genes, inflammasome activation, regulation of cell death and modification of cell signals such as NFκB and MAPK. NLR family members are important immune defense mechanisms that sense pathogen-associated molecular patterns and danger-associated molecular patterns. Accumulating evidence has shown the pivotal roles of these intracellular immune receptors/sensors, particularly the NLRP3 inflammasome, in sensing systemic metabolic perturbations. Other NLRs have recently been shown to modulate intracellular metabolic pathways that shape the phenotype of immune cells. Although the experimental evidence is far from complete, we will summarize and discuss the recent findings on the roles of NLRP3, NLRX1, and NLRC3 in metabolic reprogramming.

### NLRP3 inflammasome

As a critical component of innate immunity, the inflammasome is a large multimeric protein complex consisting of the cytosolic sensor NLR, the adapter ASC (apoptosis-associated speck-like protein containing a C-terminal caspase recruitment domain) and the effector procaspase-1 [42]. In addition, NEK7, a member of the NIMA-related kinase family, was recently identified as an NLRP3-binding component that was essential for NLRP3 inflammasome oligomerization and activation [43–45]. The NLRP3 inflammasome is the most prominent inflammasome and promotes innate and adaptive immune responses. The inflammasome is generally activated in “wo steps”. The first signal (priming) triggers the NF-κB-mediated upregulation of inactive pro-IL-1β and NLRP3 (or, in some cases, nontranscriptional priming) [42, 46], as well as posttranslational modifications, such as ubiquitylation, phosphorylation, sumoylation, neddylation, acetylation, ADP-ribosylation and nitrosylation [42, 47–54]. The second signal consists of the engagement of an NLRP3 activator and drives inflammasome assembly, proximity-induced caspase-1 autoproteolysis, and the cleavage of IL-1β, IL-18, and gasdermin D, culminating in the secretion of mature active cytokines and the induction of pyroptosis, which is a form of inflammatory programmed cell death [42, 55–60]. NLRP3 acts as a guardian of intracellular homeostasis and are activated by a variety of endogenous signals [42, 61], including the efflux of potassium or chloride ions, intracellular calcium mobilization, ROS, cytosolic mitochondrial DNA (mtDNA) and cardiolipin, mitochondrial damage, lysosomal disruption, endoplasmic reticulum (ER) stress, trans-Golgi disassembly, intracellular kinase signaling, and lipid uptake and accumulation [42, 46, 61]. The NLRP3 inflammasome participates in the progression of metabolic diseases, is activated by systemic metabolic disturbances (e.g., dyslipidemia and hyperglycemia) [62–71], and is a modifiable target in therapeutic approaches [72–77].

**Fig. 2:**
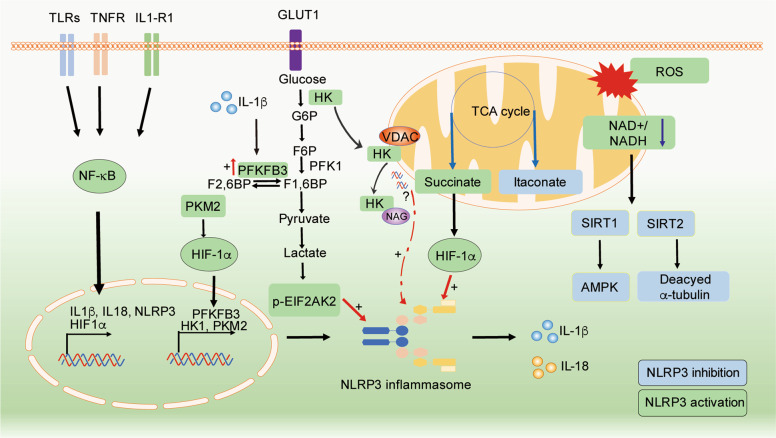
Immunometabolic regulation of NLRP3 inflammasome activity. The glycolytic cascade downstream of active PKM2 drives the production of lactate, which mediates EIF2AK2 phosphorylation and enhances IL-β-mediated PFKFB3 expression to activate NLRP3. Dimeric PKM2 interacts with HIF-1α, thus promoting *IL-β* expression and glycolysis. Bacterial NAG binds to and inhibits HK activity, which causes the dissociation of HK from VDACs and in turn releases unknown factors from mitochondria to trigger NLRP3 inflammasome activation. Aberrant mitochondrial homeostasis results in the accumulation of the TCA derivatives succinate (in green) and itaconate (in blue), which activate and suppress NLRP3, respectively. Reduced NAD^+^ availability inactivates SIRT1/2, resulting in two activation signals for NLRP3: (i) AMPK inhibition and (ii) the deposition of acetylated α-tubulin. Green: inducers of NLRP3 activity. Blue: inhibitors of NLRP3

Accumulating evidence has suggested that NLRP3 activation is tightly regulated by changes in intracellular metabolic pathways, especially within immune myeloid cells (Fig. 2). Moon et al. showed that in macrophages stimulated with LPS and ATP, HK1-dependent glycolysis was a critical step in NLRP3 activation. Glucose deprivation, glycolysis inhibition (by 2-DG treatment) or silencing *Hk1* suppressed caspase-1 activation and IL-1β secretion. Furthermore, activation of the Raptor/mTORC1 complex promoted the HK1-dependent increase in glycolytic flux to cause an inflammasome response in ATP-stimulated LPS-primed macrophages [4]. Consistently, Xie et al. [78] used pharmacologic inhibition and silencing of PKM2 and showed that PKM2-dependent glycolysis was required for the activation of NLRP3 inflammasomes after LPS-primed macrophages were treated with ATP. Mechanistically, inflammasome activation is associated with glycolysis-driven autophagy inhibition and mitochondrial ROS (mtROS) production. In addition, PKM2-driven glycolysis results in the phosphorylation of eukaryotic translation initiation factor 2 alpha kinase 2 (EIF2AK2), which functions in the antiviral response, inflammation, and immune regulation [79] and can physically interact with NLRP3, NLRP1, NLRC4, or AIM2 [80] and promote NLRP3 activation [78, 80]. Pharmacologic or genetic inhibition of PKM2 or EIF2AK2 hampers NLRP3 inflammasome activation and protects mice from lethal endotoxemia and polymicrobial sepsis [78]. Similarly, monosodium urate and calcium pyrophosphate crystals, which are linked to gout and pseudogout flares, promote glucose uptake in macrophages and synovial fluid neutrophils, triggering glycolysis-dependent NLRP3 activation [81]. A recent study showed that IL-1β drives PFKFB3 expression and glycolysis in macrophages stimulated with LPS and amyloid-β, whereas inhibiting NLRP3 or PFKFB3 activity with MCC950 [82] or 3PO, respectively, inhibits glycolytic induction in stimulated macrophages [83]. Accordingly, channeling the glycolytic end-product pyruvate toward lactic acid fermentation rather than mitochondrial pyruvate oxidation is important for optimal NLRP3 activation in response to various NLRP3 agonists in macrophages. This effect appears to depend on the lactate-mediated phosphorylation of EIF2AK2, which regulates inflammasome responses [80, 84]. These findings indicate that NLRP3 may create a metabolic loop in which glycolysis is induced upstream and downstream of NLRP3 inflammasome activation.

In contrast to these findings that support the roles of glycolytic enzymes in NLRP3 activation, other studies have reported that the disruption of glycolytic flux serves as an activating signal for NLRP3. For example, Wolf et al. showed that in LPS-primed macrophages, peptidoglycan (PGN) activates NLRP3 through the release of N-acetylglucosamine (NAG), a sugar subunit of the backbone of PGN that is produced during the lysosomal degradation of PGN. Mechanistically, both PGN and NAG inhibit the glycolytic enzyme hexokinase, thereby inducing hexokinase dissociation from the mitochondrial membrane into the cytosol. However, HK dissociation but not inhibition trigger NLRP3 inflammasome activation [85]. Moreover, although NAG treatment did not affect mitochondrial membrane integrity, it induced the cytosolic translocation of mtDNA [85], which is a known NLRP3 activator [86–88]. Accordingly, in bone marrow-derived macrophages (BMDMs), glycolytic disruption caused by the small molecule GB111-NH_2_ or infection with *Salmonella typhimurium* induced NLRP3 inflammasome formation, IL-1β secretion, and pyroptotic cell death via a mechanism involving mtROS production and impaired NADH formation mediated by glycolysis [89]. However, facilitating the TCA cycle through supplementation with the glycolytic end-product pyruvate abrogated inflammasome activation [89], indicating that the NLRP3 activating signal was downstream of glycolytic interruption. Overall, these studies indicate that while glycolysis is important for the induction of NLRP3 expression and inflammasome assembly, perturbations in the downstream TCA cycle and OXPHOS also provide the mitochondrial-derived danger signals necessary for NLRP3 activation. In addition, in contrast to inflammasome dependence on PKM2-driven glycolysis [78], the activation of PKM2 by the small molecules DASA-58 and TEPP-46 was shown to hamper the LPS-induced proinflammatory response in M1 macrophages while stimulating the secretion of the anti-inflammatory M2 IL-10 cytokine in vitro [3]. Likewise, in vivo TEPP-46 administration inhibited the secretion of IL-1β but increased IL-10 production in LPS-induced sepsis and in a *S. typhimurium* model of infection [3]. This incongruence can be explained by the fact that PKM2 exists as dimers and tetramers that have opposite effects on glycolysis. PKM2 dimers can interact with HIF-1α and promote HIF-1α transcriptional activity, leading to the expression of glycolytic enzymes, as well as *Il1b*, whereas tetramerization of PKM2, such as in response to DASA-58 and TEPP-46 [90], impairs PKM2 nuclear translocation and association with HIF-1α [3, 91, 92]. In fact, DASA-58 and TEPP-46 treatment ablate LPS-induced HIF-1α expression/stabilization and upregulation of glycolytic rates [3].

As described above, acute inflammatory signaling favors aerobic glycolysis at the expense of mitochondrial OXPHOS. This shift results in the accumulation of TCA cycle intermediates, such as succinate. Succinate is produced in mitochondria by the succinyl-CoA enzyme and is further oxidized into fumarate by the enzyme succinate dehydrogenase (ETC complex II), thereby contributing to the pool of mtROS [2, 93]. Alternatively, succinate can be exported from the mitochondria and function as an immunometabolite in the cytosol or extracellular space [94–96]. Once in the cytosol, succinate inhibits prolyl hydroxylases and thus promotes HIF-1α stabilization and activation [94, 97], resulting in the upregulation of glycolytic enzymes [3, 11, 38, 98]. In a hallmark study, O’ Neill’s group identified succinate as an intracellular innate signal that accumulates in LPS-stimulated macrophages and induces HIF-1α-driven IL-1β upregulation. These effects were strictly dependent on the metabolic rewiring triggered by TLR4 signaling upon engagement by LPS [38]. More recently, the same group revealed that another TCA-derived immune metabolite, itaconate [39], regulated IL-1β by specifically blocking NLRP3 activation. Using macrophages that lacked the enzyme IRG1, which catalyzes itaconate production, and tandem mass spectrometry, the authors showed the specificity of itaconate and its cell-permeable derivative 4-octyl-itaconate in inhibiting NLRP3 activity and the occurrence of the itaconation of cysteine C548 on the NLRP3 protein [76]. The latter process is likely responsible for impairing protein–protein interactions in the inflammasome complex. The anti-inflammatory effects of 4-octyl-itaconate have also been reported in PBMCs isolated from CAPS (cryopyrin-associated periodic syndrome) patients and in an in vivo model of peritonitis induced by monosodium urate crystals as an NLRP3 activating signal [76]. Moreover, endogenous itaconate was recently shown to accumulate in macrophages, particularly in the context of prolonged LPS stimulation, and posttranslationally modify gasdermin D, thereby impairing caspase-1 processing and late NLRP3 inflammasome activation [99]. Further reports suggest an inhibitory effect of itaconate on IL-1β secretion in activated macrophages by regulating the enzymatic activity of mitochondrial SDH and glycolytic GAPDH and fructose-bisphosphate aldolase A [37, 100–102]. Consistent with the evidence that intracellular metabolites can regulate NLRP3 activation, other derivatives of TCA cycle metabolites, such as ethyl pyruvate (EP) and dimethyl fumarate (DMF), have been shown to hamper NLRP3 activation [103–105]. This suppressive effect depends on the ability of EP to attenuate mitochondrial damage and inhibit mtDNA release into the cytoplasm and by the DMF-mediated induction of the antioxidant NF-E2-related factor 2 (Nrf2), ultimately reducing mtROS production and cytoplasmic translocation of mtDNA [103, 105].

Mitochondria are central in NLRP3 inflammasome activation: NLRP3 is recruited to mitochondria for optimal inflammasome activity [106], and inflammasome responses are largely triggered by aberrant mitochondrial homeostasis [107]. Dysfunctional mitochondria and disturbances in OXPHOS are associated not only with mtROS generation but also the decline in NAD^+^ levels, resulting in the inhibition of NAD^+^-dependent enzymes, such as sirtuins [108]. Sirtuins (SIRT1–7) are a family of evolutionarily conserved NAD^+^-dependent protein deacetylases that serve as key metabolic sensors and govern metabolic homeostasis, stress responses, and longevity [108]. Notably, decreased NAD^+^ levels have been reported in LPS-primed BMDMs [89, 109, 110], suggesting the impairment of SIRT activity in primed macrophages. Specifically, SIRT1 and NLRP3 seem to mutually regulate each other: inflammasome-activated caspase-1 can cleave SIRT1 and inhibit its activity [69, 111], whereas SIRT1 can restrain NLRP3 activation by activating the LKB1 (liver kinase B1)/AMPK pathway, thereby promoting mitochondrial biogenesis, OXPHOS and autophagy and indirectly limiting dysfunctional mitochondria [28, 61, 88]. In addition, the activity of NAD^+^-dependent SIRT2 has also been shown to modulate NLRP3 activation. Misawa *et al*. found that inducers of NLRP3 disrupted mitochondrial homeostasis and diminished intracellular NAD^+^, which consequently inhibited the SIRT2-mediated deacetylation of α-tubulin. As a result, the accumulation of acetylated α-tubulin mediated mitochondrial transport to the ER, resulting in the accumulation of NLRP3 on the ER with mitochondrial ASC [109]. Moreover, He et al. demonstrated that SIRT2 prevented inflammaging by deacetylating NLRP3 and inactivating the NLRP3 inflammasome. Instead, the acetylation of NLRP3 facilitates inflammasome assembly and enhances its activity in macrophages, underscoring the link between TCA metabolite availability and regulatory posttranslational modifications of NLRP3 [53].

NLRP3 activity can be triggered by systemic metabolic perturbations, such as increased dietary lipids, which fuel oxidative catabolic metabolism [112]. For example, Wen et al. reported that the saturated fatty acid palmitate, which is highly abundant in Western diets and in the circulation of obese diabetic individuals, can activate the NLRP3 inflammasome by suppressing AMPK activity, consequently inhibiting autophagy while increasing ROS production [62]. In addition, AMPK restrains inflammation by negatively regulating mTOR complex I, which is accompanied by the suppression of protein and lipid synthesis required for mounting a proper inflammatory response [12].

While the intracellular accumulation of cholesterol and saturated fatty acids is regarded as a trigger of inflammasome assembly [62, 64, 65], polyunsaturated FAs, such as the ω-3 FA docosahexaenoic acid, attenuate NLRP3-driven inflammation [113, 114]. In contrast to FAS, which accompanies proinflammatory functions, FAO is typical of type 2 immunity and anti-inflammatory functions [2] and produces NADH, FADH_2_, and acetyl coenzyme A, which are further used to generate ATP or ketone bodies under starvation conditions [1]. Interestingly, two studies showed that the main ketone metabolite beta-hydroxybutyrate is a potent inhibitor of NLRP3 priming and assembly in macrophages and neutrophils [77, 115]. However, another study by Moon et al. indicated that FAO could promote NLRP3 assembly, while pharmacological inhibition or genetic ablation of the enzyme NADPH oxidase 4 (NOX4) reduced FAO rates and NLRP3 activation [116]. In contrast, in a murine stroke model, administration of the NOX inhibitor apocynin resulted in neuroprotective and anti-inflammatory effects, and there was a reduction in the expression of NLRP3, ASC, caspase-1, IL-18, and IL-1β [117]. These findings suggest that specific enzymes and metabolic pathways differentially govern the assembly and activation of the NLRP3 inflammasome depending on the location and underlying pathology.

Overall, these studies highlight the intricate relationship between the NLRP3 inflammasome and metabolism. Subtle alterations in one metabolic pathway can perturb associated metabolic circuits, leading to the accumulation of specific endogenous metabolites or dangerous molecules that in turn activate NLRP3 and induce inflammation (Fig. 2).

### NLRX1

NLRX1 (nucleotide-binding domain and LRR-containing protein X, also known as CLR11.3, NOD5, or NOD9) was first discovered as a mitochondria-associated NLR that possesses a mitochondrial targeting sequence (MTS) encoded in the first 39 amino acids of the protein. NLRX1 consists of 975 amino acids and is ubiquitously expressed in mammalian tissues. It contains two of the three typical NLR domains (a central NACHT domain and a carboxy-terminus LRR domain) and an unconventional N-terminal domain that gives rise to the ‘X’ in the nomenclature [118–120]. NLRX1 was first shown to negatively regulate mitochondrial antiviral signaling protein (MAVS)-mediated type I IFN and NF-κB signaling by interacting with MAVS through its LRR domain at the mitochondrial outer membrane [119]. Furthermore, NLRX1 has also been shown to directly bind to RNA [121–123]. NLRX1 is also present in the inner mitochondrial membrane and within the matrix [118, 120]. Internalized NLRX1 interacts with the protein UQCRC2 in the ETC and regulates the production of ROS in mitochondria [118]. Hence, it is possible that the distribution of NLRX1 in distinct cellular compartments allows it to interact with multiple molecules of cellular pathways to exert its diverse effects and modulate immune responses. NLRX1 has been linked to the regulation of the host innate immune response in the context of pathogen sensing, inflammation, ROS production, ER stress, and autophagy [121, 124–134]. One consistency is that many studies have shown that NLRX1 attenuates diseases, including chronic obstructive pulmonary disease (COPD) [135], autoimmune diseases [136–139], and cancer [140–144]. In cancer, NLRX1 has been shown to downregulate key cytokines that are important in cancer progression, such as tumor necrosis factor (TNF) and interleukin (IL)-6. Accumulating evidence indicates that NLRX1 has multiple functions [145, 146]. In this review, we will focus on its role in regulating metabolic reprogramming.

In the immunometabolism field, NLRX1 is a bridge between inflammation and metabolism. In cancer cells, the removal of extracellular glucose or the addition of the glycolysis inhibitor 2-deoxyglucose (2-DG) significantly reduced NLRX1 expression in both primary- and SV40-transformed MEFs. This result indicates that the expression of NLRX1 is glucose-regulated [147]. In addition, NLRX1 was also shown to regulate glycolysis. In one study, knockdown of NLRX1 in HeLa cells reversed the acidification of the culture medium, suggesting that NLRX1 may contribute to the metabolic switch to glycolysis in tumor cells [144]. NLRX1 also regulates the activities of mitochondrial complex I and III to maintain ATP levels in the presence of TNF-α and promote the metabolic switch toward aerobic glycolysis. Specifically, NLRX1 depletion in MDA-MB-231 cells (a triple-negative breast cancer cell line) decreased the organization and activity of OXPHOS complexes, affecting OXPHOS-dependent cell proliferation and the migration of triple-negative breast cancer cells. The loss of NLRX1 in MDA-MB-231 cells further impaired lysosomal function and mitophagy-mediated turnover of damaged mitochondria in the presence of TNF-α [148]. These studies suggest that NLRX1 functions as a critical mitochondrial protein to maintain mitochondrial metabolism for energy homeostasis and lysosomal function to regulate the invasion and metastasis capabilities of metastatic breast cancer [144, 148].

Other studies have linked NLRX1 to fatty acids and amino acid metabolites. For example, compound libraries were screened, and different lipids, such as coenzyme A-containing fatty acids and sterols, were identified that could bind to the LRR domain of NLRX1. Moreover, punicic acid (PUA), a polyunsaturated fatty acid, or docosahexaenoic acid (DHA), exerted anti-inflammatory effects and suppressed NF-κB activity in an NLRX1-dependent manner in LPS-activated BMDMs. In contrast, PUA treatment did not ameliorate experimental colitis in *Nlrx1*^−*/*−^ mice compared to wild-type mice. The study further indicated that the interaction of NLRX1 and PUA was necessary for modulating mucosal immune responses and relieving inflammation in the gut [149]. Another study showed that NLRX1 protected against mitochondrial damage and oxidative stress in kidney epithelial cells and that the loss of NLRX1 promoted oxygen consumption and oxidative stress, disturbed mitochondrial morphology, and subsequently increased ROS production and apoptosis in tubular epithelial cells during renal ischemia-reperfusion injury. The NLRX1-mediated loss of ROS in tubular cells contrasts with the findings of an earlier study suggesting that NLRX1 induced ROS; hence, the effect of NLRX1 on ROS may be dependent on the experimental conditions and cell types. Of note, this study also found that the polyunsaturated fatty acid DHA, but not the saturated fatty acid palmitate, prevents apoptosis during reoxygenation in an NLRX1-dependent fashion, confirming the finding shown by Lu et al. that polyunsaturated fatty acids can modulate NLRX1 function [150].

Leber et al. showed that *Nlrx1*^*−/−*^ intestinal epithelial cells (IECs) showed increased expression of genes associated with amino acid metabolic pathways, specifically glutamine metabolism (*Glud1*, *Got1*, and *Gpt*), based on RNA-sequencing data. Moreover, *Nlrx1*^*–/–*^ intestinal organoids have significantly increased activity of glutamate dehydrogenase (GDH), which catalyzes the reversible conversion of the oxidative deamination of glutamate to α-ketoglutarate and ammonia while turning NAD(P)^+^ to NAD(P)H [151] and decreases NAD^+^ levels without changing NADH levels, indicating altered cycling or consumption of NAD^+^ in these cells. Interestingly, oral glutamine supplementation altered the microbiome composition and alleviated the severity of inflammatory bowel disease in *Nlrx1*^−/−^ mice [152]. This finding provides a link between NLRX1 and glutamine metabolism and suggests that the administration of specific amino acids, such as glutamine, can ameliorate intestinal inflammation by modulating the microbiome in an NLRX1-dependent manner.

Interestingly, NLRX1 is also expressed by CD4^+^ T cells, and the loss of NLRX1 results in increased disease severity and increased numbers of Th1 and Th17 cells producing inflammatory cytokines (IFN-γ, TNF, and IL-17) in dextran sodium sulfate-induced (DSS) colitis. *Nlrx1*^−/−^ CD4+ T cells show altered metabolic behavior with increased expression of the genes *Cpt1a*, *Fabp4*, and *Glut1*, which are responsible for the uptake and utilization of glucose and fatty acids. Likewise, the loss of NLRX1 in CD4^+^ T cells increases the rate of incomplete FAO and enhances glycolysis in Th17 cells [153]. To evaluate NLRX1 as an immunometabolic regulator of inflammation, the small molecule NX-13 (1,3,5-tris(6-methylpyridin-2-yloxy)benzene was shown to bind to NLRX1. The drug was delivered orally, exhibited increased local concentrations throughout the gastrointestinal tract, including the distal parts of the colon, and selectively targeted and activated NLRX1 in CD4^+^ T cells in IBD. NX-13 treatment in vitro decreased the differentiation of CD4^+^ T cells into Th1 and Th17 subsets increased OXPHOS and decreased NF-κB activation and ROS. NX-13 treatment in vivo reduced Th1 and Th17 subsets, enhanced Treg cells, and reduced neutrophils in high-dose DSS-induced colitis. In addition, NX-13 decreased inflammatory cytokines such as IL-8, MCP1, and IL-6, as well as NF-κB and ROS production in PBMCs from moderate-to-severe ulcerative colitis patients. These studies suggest that NX-13 is a promising NLRX1 agonist and a translational medicine candidate for treating a number of NLRX1-attenuated inflammatory conditions [154, 155].

In addition to glucose metabolism, NLRX1 also regulates fatty acid metabolism. *Nlrx1*^*−/*−^ mice are protected against high-fat diet-induced metabolic syndrome, kidney dysfunction and the progression of nonalcoholic fatty liver disease (NAFLD). The loss of NLRX1 in hepatocytes also leads to increased FAO and decreased steatosis. Hence, NLRX1 controls nonimmune parenchymal hepatocyte energy metabolism by restricting mitochondrial fatty acid-dependent OXPHOS and enhancing glycolysis [156]. Likewise, *Nlrx1*^*−/*^^−^ mice are protected against high-fat diet-induced pancreatic lipid accumulation and hyperglycemia [157].

**Fig. 3:**
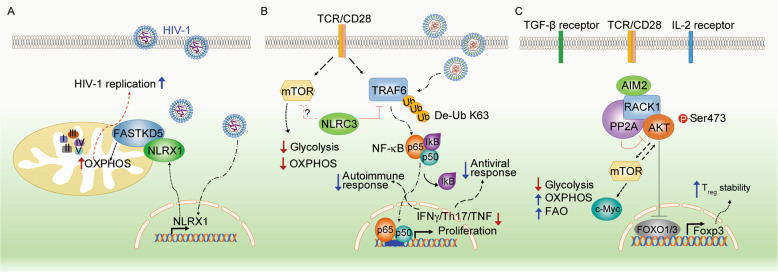
Overview of intracellular innate immune receptors in T cell metabolic reprogramming. **A** HIV-1 infection increases the expression of NLRX1, which directly associates with FASTKD5 to promote OXPHOS and viral replication. **B** NLRC3 in T cells acts as an intrinsic attenuator that regulates T cell signaling pathways and metabolic progress to restrict autoimmune and virus-specific CD4+ T cell responses. **C** AIM2 facilitates the interaction between RACK1 and PP2A phosphatase, causing the dephosphorylation of AKT to restrain the activity of the mTOR pathway, thereby promoting Foxp3 expression and T_reg_ cell stability

Singh et al. showed that NLRX1 is associated with Fas-activated serine-threonine kinase family protein-5 (FASTKD5) through its LRR domain and colocalized with mitochondrial RNA granules in the mitochondrial matrix. FASTKD5 negatively regulates the processing of mitochondrial transcripts to modulate the activity of complex-I and complex-IV and respiratory supercomplex formation [158]. An earlier report also identified FASTKD5 as a putative NLRX1 interaction partner using overexpressed NLRX1 as the bait for immunoprecipitation followed by mass spectrometry [159]. The interaction of NLRX1 and FASTKD5 has been further confirmed in mitochondria and has been shown to negatively regulate RNA processing, OXPHOS activity, and mitochondrial ribosome biogenesis and translation [160]. The connection of NLRX1 with FASTKD5 is also relevant to T cells, and the association of NLRX1 and FASTKD5 has been shown to enhance the expression of mitochondrial respiratory complex components in human CD4^+^ T cells and promote OXPHOS and glycolysis [161]. The study showed an essential role of OXPHOS in promoting HIV-1 replication in human CD4^+^ T cells, which is achieved in an NLRX1- and FASTKD5-dependent manner. The study underscores the importance of NLRX1-mediated regulation of immunometabolism, which is relevant for controlling HIV-1 in CD4^+^ T cells [161] (Fig. 3A) and provides a rationale for the therapeutic targeting of NLRX1 or OXPHOS for HIV treatment.

In addition, NLRX1 has been shown to protect experimental autoimmune encephalomyelitis (EAE), a commonly used experimental animal model of human multiple sclerosis (MS), by attenuating microglial activation and reducing encephalitogenic T cells [138, 139, 162]. *Nlrx1* mRNA expression is significantly increased in CD14^+^ PBMCs in relapsing-remitting MS patients, and the identification of rare NLRX1 mutations in MS patients, such as p.Lys172Asn, p.Glu192Ter, and p.Arg860Trp links NLRX1 genetic variants to the incidence of MS in humans. These studies indicate that NLRX1 may be a therapeutic target for MS. A recombinant protein (dNP2-LRR), which is consistent with the blood–brain barrier permeable peptide dNP2 [163] and the LRR domain of NLRX1, alleviates EAE [164]. In summary, these studies provide a perspective on targeting NLRX1 in not only pathogen infection but also autoimmune diseases. Going forward, it will be intriguing to examine whether metabolic modulators that have shown efficacy in modulating NLRX1-dependent functions, such as NX-1 or rapamycin, can be used to mitigate diseases such as MS or COPD.

### NLRC3

NLRC3 (NLR family CARD-containing 3) is also named CLR16.2 or NOD3 and consists of N-terminal caspase activation and recruitment domain, a central nucleotide-binding domain, and a C-terminal LRR domain [165]. NLRC3 was first identified in human Jurkat T lymphocytes, and its expression is downregulated rapidly upon stimulation with anti-CD3/anti-CD28 antibodies or treatment with PMA/ionomycin. This finding suggests that NLRC3 functions as a suppressor of T cell activation [166]. NLRC3 is predominantly expressed in both human and murine immune tissues and cells [166]. In addition, as an innate immune receptor, NLRC3 negatively regulates LPS-induced NF-κB activation downstream of TLRs by interacting with TNF receptor-associated factor 6 (TRAF6) and further altering the ubiquitination state of TRAF6 in macrophages [167]. The loss of NLRC3 was found to enhance the innate immune response to cytosolic DNA, DNA viruses, and cyclic di-GMP [168]. NLRC3 interacts with both stimulators of interferon genes (STING), which is a DNA sensor, and the protein kinase TBK1 and impairs the STING–TBK1 interaction, resulting in the subsequent reduction in downstream type I interferon production and NF-κB activation [168]. Furthermore, NLRC3 binds HSV-60 double-stranded DNA (dsDNA) mainly through its LRR domain. Viral DNA binding to NLRC3 increases its ATPase activity, which is needed to facilitate the release of STING and TBK1 and then mediate the downstream IFN-I response [123]. Tocker et al. [169] identified a scaffolding protein, IQGAP1, that specifically associates with NLRC3 and disrupts the NLRC3–STING interaction to regulate the type I IFN pathway in the cytosol of human monocytic and epithelial cells.

In addition to pathogen sensing, the expression of NLRC3 is reduced in the colorectal tumor tissues of patients compared to healthy controls [170]. Karki et al. [170] further showed that *Nlrc3*^*−/−*^ mice are more susceptible to colitis and colorectal tumorigenesis than wild-type mice and that the oncogenic inhibitory effect of NLRC3 is more dominant in IECs. At the immunometabolism level, *Nlrc3*^*−/−*^ mice show increased phosphorylation of S6 kinase, 4E­BP1, and AKT at Ser473, all of which are downstream targets of mTOR. Mechanistically, NLRC3 associates with the p85 subunit of PI3K, disrupting the interaction between the PI3K p85 and p110α subunits and reducing the activity of PI3K p85 itself. Additionally, deletion of the CARD, NACHT, or LRR domains of NLRC3 impairs the ability of NLRC3 to interact with either the p85 or p110α subunit of PI3K. These studies demonstrated that NLRC3 restricts the PI3K–mTOR axis during colon tumorigenesis [170, 171]. The PI3K–mTOR axis has been shown to be a central integrator of immune cell metabolism and regulates glucose and fatty acid metabolism during homeostasis and diseases [172–175]. In CD4^+^ T cells, NLRC3 attenuates interferon-γ and TNF expression by CD4+ T cells and reduces T helper 1 (Th1) and Th17 cell proliferation. Moreover, the loss of NLRC3 in T cells results in improved protection against lymphocytic choriomeningitis virus infection but worsens EAE and exacerbates Th1 and Th17 responses during *M. tuberculosis* infection [176, 177]. Interestingly, *Nlrc3*^*–/–*^ CD4+ T cells showed increased phosphorylation of 4E-BP1, which lies downstream of mTOR, and increased glycolysis and OXPHOS occurred upon TCR stimulation, enhancing the proliferation and cytokine production in *Nlrc3*^*−/−*^ CD4+ T cells. Mechanistically, NLRC3 in T cells interacts with TRAF6 and negatively regulates its K63-linked ubiquitination to reduce downstream NF-κB signaling and diminishes metabolic pathways to attenuate CD4+ T cell activation and proliferation [177] (Fig. 3B). In addition, a recent study described the effect of NLRC3 on platelet‐derived growth factor (PDGF)‐induced proliferation in pulmonary artery smooth muscle cells (PASMCs) by inhibiting the PI3K‐mTOR pathway [178]. Specifically, PDGF induced abnormal PASMC proliferation, and this effect was inhibited by both a PI3K inhibitor (Ly294002) and an mTOR inhibitor (rapamycin). Overexpression of NLRC3 suppressed the expression of PI3K and mTOR in PDGF-stimulated PASMCs. This study suggests that the NLRC3/PI3K/mTOR pathway plays a critical role in PASMC proliferation. In summary, these studies revealed the impact of NLRC3 on metabolism in different cell types to control cell proliferation and activation by modulating PI3K–mTOR signaling and NFκB-mediated pathways.

## Absent in melanoma 2

AIM2 (absent in melanoma 2) is a non-NLR molecule belonging to the AIM2-like receptor (ALR) family and is a DNA receptor that triggers inflammasome activation [179–182]. AIM2 consists of C-terminal HIN-200 and N-terminal pyrin (PYD) domains and is a cytosolic DNA receptor that can directly bind to dsDNA via the HIN-200 domain. Its PYD domain interacts with the PYD domain of the inflammasome adapter protein ASC (ASC-like protein containing a carboxy-terminal CARD). The caspase activation and recruitment domain (CARD) of ASC bind to the CARD of procaspase-1 to form the AIM2 inflammasome, which further induces caspase-1 activation, leading to downstream IL-1β and IL-18 maturation and gasdermin D cleavage [183]. AIM2 recognizes dsDNA in a sequence- and structure-independent but the length-dependent manner, which requires a minimum of 80 base pairs for optimal inflammasome activation [184]. Under steady-state conditions, AIM2 is in an autoinhibitory confirmation due to the interaction of the PYD and HIN domains, and this state is altered by dsDNA binding to the HIN domain, which further recruits ASC to interact with the free PYD domain and initiates oligomerization and inflammasome activation [184, 185]. The importance of the inflammasome-dependent role of AIM2 is observed in the sensing of microbial DNA during infectious diseases and in tumorigenesis and several inflammatory and autoimmune diseases, such as atherosclerosis, neuroinflammation, psoriasis, dermatitis, arthritis, SLE, and colitis [186–188]. Next, we will highlight the evidence linking AIM2 to immunometabolism.

Sepsis is an overreactive host immune response to microbial infection that results in dysregulated systemic inflammation accompanied by the secretion of multiple proinflammatory mediators, such as TNF, IL-1β, and high mobility group Box 1 (HMGB1) [189]; sepsis has also been linked to immunometabolism in macrophages. Previous reports have shown that the M2 isoform of pyruvate kinase muscle 2 (PKM2) regulates glycolysis to promote IL-1β and HMGB1 release in LPS-stimulated macrophages during sepsis pathogenesis [3, 190]. As mentioned in the NLRP3 section, Xie et al. showed that PKM2-mediated metabolic programming (glycolysis) promotes not only NLRP3 but also AIM2 inflammasome activation by producing lactose to modulate EIF2AK2 phosphorylation in macrophages during sepsis. Pharmacologic inhibition of the PKM2-EIF2AK2 pathway can reduce inflammasome activation and protect mice from endotoxemia and sepsis. In addition, the loss of PKM2 suppresses inflammasome activation, and the specific deletion of PKM2 in myeloid cells protects septic mice against death in response to the AIM2 inflammasome [78]. Thus, this study provides a rationale for the therapeutic targeting of the PKM2-EIF2AK2 axis to control inflammasome by modulating immunometabolism. In addition to PKM2-mediated glycolysis in macrophages, which promotes both NLRP3 and AIM2 inflammasome activation [78], Cho et al. recently demonstrated a novel mechanism by which activation of the AIM2 inflammasome links glucose transporter 1 (GLUT1)-mediated glycolysis to regulate the acute exacerbation of lung fibrogenesis during bacterial infection [191]. Myeloid cell-specific *Glut1* knockout (LysM-Cre-*Glut1*^*fl/fl*^) results in reduced morbidity and collagen levels in bleomycin-induced lung fibrosis upon *Streptococcus pneumoniae* infection. It also results in reduced activation of the AIM2 inflammasome by poly(dA:dT) in *Glut1-*deficient cells in vitro [191].

In addition to innate immunity and inflammation, recent studies have revealed that AIM2 plays an essential role in cancers or autoimmune diseases in an inflammasome-independent manner by altering PI3K–AKT–mTOR signaling and immunometabolism. AIM2 was originally discovered to be a tumor suppressor gene in human melanomas [192], and reduced AIM2 expression was associated with poor prognosis in patients and AIM2 mutations in human colorectal tumors [193–195]. In colorectal cancer (CRC), Wilson et al. and Man et al. demonstrated that AIM2 suppressed azoxymethane (AOM)/DSS-induced colitis-associated cancer, as well as spontaneous CRC, in a mouse model [196, 197]. These two independent studies showed increased tumor burdens and shortened colon lengths in *Aim2*^*−/−*^ mice compared to wild-type mice, while the inflammasome-dependent cytokines IL-1β and IL-18 were intact in the *Aim2*^*−/−*^ colon, indicating that AIM2 plays an inflammasome-independent role in the development of colon cancer. Mechanistically, AIM2 restricts proliferation but promotes cell death in colon progenitors by inhibiting AKT activation, which is the central molecule in the PI3K–mTOR pathway [196, 197]. Furthermore, AIM2 associates with and restricts the activity of DNA-dependent protein kinase (DNA-PK), a PI3K-related family member, to suppress AKT activation [196]. Similar to the finding in CRC, AIM2 expression in hepatocellular carcinoma (HCC) patient tissues was negatively correlated with HCC progression [198, 199]. In HCC cells, AIM2 overexpression inhibits HCC cell proliferation and invasion by suppressing the mTOR-S6K1 axis in an inflammasome-dependent manner [198]. The PI3K–AKT–mTOR signaling pathway plays a critical role in providing nutrients for the growth of normal immune cells and tumor cells by promoting anabolic processes (glycolysis) and regulating other metabolic processes, such as protein and lipid synthesis, OXPHOS, and autophagy, which is a conserved catabolic process [175, 200]. Hence, these studies link AIM2 with the PI3K–AKT–mTOR signaling pathway in cancer cells.

Importantly, two independent groups recently reported that AIM2 regulates AKT activation in immune cells during EAE. Ma *et al*. found that AIM2 negatively regulated EAE progression in an inflammasome-independent manner in microglia in both the A and B subtypes of EAE [201]. MOG-induced EAE can be separated into the A and B subtypes based on the use of low or high concentrations of complete Freund’s adjuvant [202]. Similar to that in tumor cells, AIM2 limits antiviral pathway-related inflammation by interacting with DNA-PK to dissociate the DNA-PK-AKT3-IRF3 complex [201]. In addition to being an innate inflammasome sensor that regulates the behavior of innate immune cells or epithelial cells during infection or inflammation, the vital role of AIM2 in controlling the function of regulatory T (Treg) cells in a T cell-intrinsic but inflammasome-independent manner has been reported in EAE and T cell-mediated colitis [203]. In contrast to previous reports showing that NLRP3, ASC, and caspase-1 exacerbate EAE [204–207], AIM2 mitigates EAE without affecting IL-1β or IL-18 but increases the number of IL-17A-producing CD4^+^ T cells and decreases Foxp3^+^ Treg cells in the spinal cord. Interestingly, AIM2 is highly expressed by both human and murine Treg cells induced by TGFβ, and its promoter is occupied by RUNX1, ETS1, BCL11B, and CREB, which are transcription factors that are associated with Treg cells. Both the adaptive transfer of *Aim2*^*–/–*^ Treg cells in T cell-mediated colitis and the specific deletion of Aim2 in Treg cells show that AIM2 is required for Treg cell stability and control of autoimmune diseases [203]. Additionally, *Aim2*^*−/*−^ Treg cells have increased glycolysis but reduced lipid OXPHOS and enhanced AKT-mTOR signaling, which indicates that AIM2 is essential in regulating Treg cell metabolism. Mechanistically, AIM2 associates with RACK1, which is a scaffold protein that forms a complex with PP2A and AKT, to dephosphorylate AKT and reduce AKT-mTOR signaling [203] (Fig. 3C). This study uncovered the functions of AIM2 in regulating immunometabolism in adaptive immune cells and suggests a unifying role of AIM2 in interacting with the AKT-mTOR metabolic pathway in many cell types. Thus, these studies show the unexpected role of intracellular innate immune sensors/receptors in the regulation of T cell immunometabolism (Fig. 3).

## Stimulator of interferon genes

STING (also known as TMEM173, MITA, MPYS, and ERIS) [208–211] is a universal receptor for cyclic dinucleotides, including the bacterial second messengers cyclic di-AMP, cyclic di-GMP and 3′,3′-cGAMP. In metazoans, cyclic GMP-AMP synthase (cGAS) lies upstream of STING and can bind dsDNA, resulting in 2′,3′-cGAMP synthesis. These cyclic dinucleotides bind to STING at the ER and promote STING trafficking from the ER to perinuclear puncta. During this process, STING recruits TANK binding kinase 1 (TBK1), which phosphorylates the transcription factor interferon regulatory factor 3 (IRF3), resulting in the production of type I interferons (IFNs). Recently, several studies have linked immunometabolic pathways and STING activation, which is the focus of the following discussion.

**Fig. 4:**
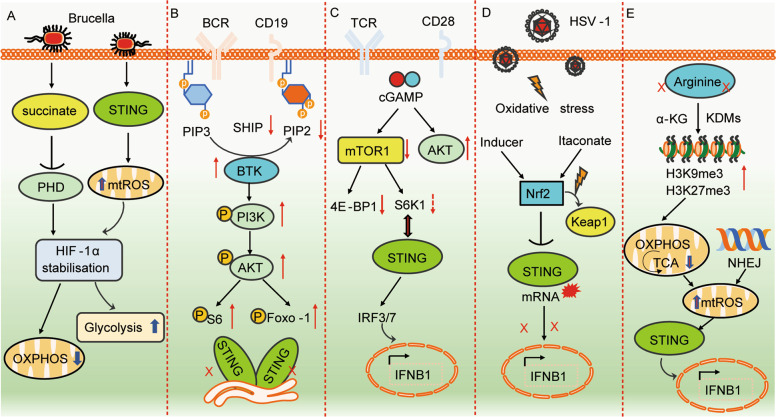
STING-mediated PI3K–AKT–mTOR signaling and activation during metabolic reprogramming. **A** STING regulates metabolic reprogramming in macrophages via HIF-1α during *B. abortus* infection. **B** STING interferes with BCR activation and negatively regulates CD19- and Btk-mediated PI3K signaling. **C** Reciprocal regulation of STING and TCR stimulation by mTORC1 leads to IFN-I production. **D** Nrf2 negatively regulates STING-mediated metabolic reprogramming in response to HSV-1 viral infection. **E** Arginine starvation induces STING activation through nuclear DNA damage via epigenetic silencing of metabolic and DNA repair genes

In myeloid cells, STING activation repolarizes M2 macrophages to M1-like macrophages, which are inflammatory and undergo metabolic reprogramming [212–214]. This repolarization of M2 macrophages to M1 macrophages occurs during *Brucella abortus* infection, and an impaired TCA cycle in M1-like macrophages drives succinate accumulation. The accumulation of succinate suppresses prolyl hydroxylase (PHD) activity, leading to HIF-1α stabilization. STING activation in M1 cells also causes increased mtROS, which similarly stabilize HIF-1α. HIF-1α then alters immunometabolism by reducing OXPHOS and increasing glycolysis (Fig. 4A). During *M. tuberculosis* infection, STING and downstream type I IFN cause metabolic reprogramming characterized by reduced glycolytic activity and OXPHOS and increased mitochondrial damage characterized by mtROS [215]. In addition to these links between STING and metabolic effectors, the mTOR downstream signal S6 kinase 1 (S6P1) forms a complex with STING and TBK1 in adenovirus-infected dendritic cells to activate IRF3 [216]. Viruses use these pathways for immune evasion. For example, the Poxviral F17 protein sequesters Raptor and Rictor in the Golgi to block cGAS-STING signaling [217].

STING also affects immunometabolism in adaptive immune cells. B cells from systemic lupus erythematosus (SLE) patients have highly activated BCRs, and STING is thought to attenuate the pathogenesis of this disease because STING expression is low in SLE patients compared to controls. In support of this conclusion, *Sting*-KO mice have increased numbers of splenic marginal zone (MZ) and germinal center (GC) B cells [218] and exhibit greater pathogenesis than WT mice. Btk-PI3K signaling plays an important role in MZ B cell homeostasis, and *Sting*-KO mice show enhanced BTK but reduced inhibitory phosphatase SHP-1 levels. Furthermore, activated pPI3K, pAkt pFoxo-1/Foxo-1, and pS6/S6 were all increased in *Sting*-KO B cells. However, in one study, STING did not alter PI3K recruitment or activity but directly affected the activity of PI3K by reducing PI(3,4)P2 levels. Furthermore, PI3K inhibitors significantly decreased the colocalization of the BCR and STING. These studies suggest the bidirectional regulation of PI3K- and STING-mediated BCR signaling (Fig. 4B).

STING is expressed by T cells, resulting in the induction of type I IFN, reduced cell proliferation and increased cell death, although the latter is not observed in vivo [219]. cGAMP inhibits CD4^+^ T cell proliferation, resulting in a sustained G0–G1 phase of the cell cycle, which is accompanied by reduced activation of mTOR1, S6K1, and 4E-BP1 [220]. RNA profiling and pathway analysis indicated that cGAMP reduces lipid synthesis-related genes that are regulated by mTORC1. Using gene deletion strains, it was found that the STING-activated IRF3/7 pathway suppressed the reduction in mTORC1 in T cells and T cell proliferation. Conversely, mTORC1 activation is needed for STING-induced type I interferon production [220]. Blocking mTOR1 with rapamycin or deleting Raptor abolished cGAMP-mediated IFN-I production in activated CD4^+^ T cells. Collectively, these findings indicate a regulatory circuit in which STING activation by cGAMP attenuates mTOR signaling, leading to reduced T cell proliferation, while mTORC1 activation via S6K1 is required for STING-mediated IFN-I production (Fig. 4C)

In other studies, glutathione peroxidase 4 (GPX4), an enzyme that balances oxidation and reduction reactions, was shown to mediate STING activation during viral infection [221]. GPX4 inhibitors can suppress IFN-β mRNA and protein expression during HSV-1 infection in murine peritoneal macrophages and human THP1 cells [221]. *Gpx4* conditional deletion results in an attenuated antiviral innate immune response due to enhanced cellular lipid peroxidation, which inhibits the cGAS-STING pathway. Increased lipid peroxidation causes the carbonylation of STING, which reduces its trafficking to the ER and blocks STING activation because STING localization to the ER is an essential step for its activation.

Others have found a link between the TCA cycle-derived metabolite itaconate (4-octyl-itaconate, 4-OI) and STING expression. Itaconate can activate the nuclear transcription factor Nrf2 (nuclear factor erythroid-derived 2-like 2) and reduce STING mRNA levels by inducing mRNA instability [222]. In addition to its in vitro relevance, itaconate can suppress STING expression and type I IFN in cells from patients with the interferonopathy and STING-associated autoimmune disease SAVI (STING-associated vasculopathy with onset in infancy) (Fig. 4D). Interestingly, another group showed that nitro-fatty acids formed by the addition of nitrogen dioxide to unsaturated fatty acids inhibit STING palmitoylation and mitigate type I interferon production in SAVI-derived fibroblasts [223].

Finally, a recent report showed a link between arginine metabolism and STING [224]. Arginine is one of three amino acids that can activate mTOR, and some cancer cells are deficient in arginine synthesis. Arginine starvation causes the depletion of α-ketoglutarate and the inactivation of histone demethylases, resulting in the silencing of genes involved in OXPHOS and DNA repair. As a result of increased DNA damage and an increase in cytosolic DNA due to arginine starvation, cGAS-STING is activated, followed by the induction of type I IFN (Fig. 4E).

Aside from metabolic reprogramming at the cellular level, STING plays an important role in several metabolic diseases, as shown in these reviews [225, 226]. STING exacerbates NAFLD and nonalcoholic steatohepatitis (NASH) disease models, and STING expression is higher in parenchymal liver cells from patients with NAFLD than in those from controls [227]. In mouse models of NASH and NAFLD, STING deficiency results in attenuated hepatic fibrosis, steatosis, and inflammation [228]. Mechanistically, NAFLD and obesity can result in increased release of oxidative mtDNA into the cytosol, which causes STING activation. Others observed obesity-induced cytosolic mtDNA release, which triggers the cGAS-STING pathway [229, 230]. The mitochondrial protein disulfide-bond A oxidoreductase-like protein (DsbA-L) blocks cGAS-STING by preventing mtDNA leakage and increases phosphodiesterase PDE3B/PDE4, cAMP, and PKA signaling to promote thermogenesis in adipocytes. These findings suggest that blocking the cGAS-STING pathway represents a new therapeutic approach for multiple metabolic diseases.

## Concluding remarks and future prospective

Our understanding of the mechanisms governing immunometabolism has been greatly expanded in the last few years, and the studies described in this review emphasize the emerging recognition of crosstalk between intracellular innate immune receptors/sensors and metabolic pathways in shaping inflammatory responses and impacting the course of inflammatory diseases. A deeper knowledge of this intricate crosstalk can lead to novel therapeutic strategies targeting immunometabolic circuits for the treatment of metabolic and inflammatory diseases. Examples of currently approved anti-inflammatory therapies that target metabolism include metformin (targeting mTOR, used for the treatment of type 2 diabetes), rapamycin (targeting AMPK and ETC complex I, used as an immunosuppressive drug), and DMF (antioxidant and enhancer of mitochondrial respiration) [231]. However, to advance our knowledge and future therapies, it is crucial to understand the spatiotemporal immunometabolic adaptations during the course of diseases and to resolve the heterogeneity of the immunometabolic networks at the single-cell level. Great progress in single-cell technologies, such as single-cell RNA sequencing, mass cytometry, Met-Flow (flow cytometry to detect metabolic enzymes), digital spatial transcriptomic, and Cellular Indexing of Transcriptomes and Epitopes by Sequencing (CITE sequencing), can provide the required platforms for addressing these important questions [232, 233]. Using these new approaches to obtain a deeper understanding of immunometabolism mediated by innate immune receptors will greatly advance our appreciation of this still-nascent field.
